# Phenolic Content, Antioxidant Activity, Phytochemical Diversity, and Toxicological Potential of *Tetradenia riparia* (Hochst.) Codd Leaves

**DOI:** 10.1002/cbdv.202503506

**Published:** 2026-05-09

**Authors:** Caroline dos Santos Giuliani, Carla Andressa Almeida Farias, Patrícia Kolling Marquezan, Karoline Longo de Aguiar, Elize Musachio, Andreara Rodrigues dos Reis, Nathália Cardoso de Afonso Bonotto, Fernanda Barbisan, Milene Teixeira Barcia, Ana Lúcia Souza Silva Mateus, Aline Sobreira Bezerra, José Laerte Nörnberg

**Affiliations:** ^1^ Department of Food Technology and Science Federal University of Santa Maria Santa Maria Rio Grande do Sul Brazil; ^2^ Department of Microbiology and Parasitology Federal University of Santa Maria Santa Maria Rio Grande do Sul Brazil; ^3^ Department of Pathology, Biogenomics Laboratory Federal University of Santa Maria Santa Maria Rio Grande do Sul Brazil; ^4^ Department of Statistics Federal University of Santa Maria Santa Maria Rio Grande do Sul Brazil

**Keywords:** antioxidant capacity, bioactive compounds, medicinal plant, toxicological evaluation

## Abstract

*Tetradenia riparia* (Hochst.) Codd is a medicinal plant widely used in traditional medicine. However, comprehensive studies integrating its chemical composition, antioxidant capacity, and toxicological safety are still limited. This study aimed to characterize the phenolic content, antioxidant activity, phytochemical diversity, and toxicological potential of *T. riparia*. The plant material was lyophilized to obtain a homogeneous sample, which was directly used for proximate composition and fatty acid profile. Aqueous extracts prepared from the lyophilized material were employed for the determination of total phenolic content, antioxidant activity by the oxygen radical absorbance capacity (ORAC) assay, toxicological evaluations, and phenolic profiling by HPLC‐ESI‐QqQ‐MS/MS. The chemical analyses revealed a diverse phenolic composition, predominantly composed of phenolic acids and flavonoids, which was associated with relevant antioxidant capacity. Toxicological assessments using human fibroblasts (HFF‐1) and genotoxicity assays indicated low toxicity at lower concentrations, supporting the safe use of the species under the evaluated conditions. Overall, these findings provide an integrated chemical and biological assessment, contributing novel data on the phytochemical composition and safety profile of *T. riparia*.

## 1 Introduction

The field of natural products stands out as one of the most promising areas of scientific research, attracting interest from various industrial sectors [1]. Natural products are recognized as important sources of bioactive compounds. However, their safety and efficacy depend on factors such as chemical composition, dosage, preparation methods, and toxicological profile, rather than on their natural origin alone. The growing demand for natural ingredients has encouraged the exploration of new plant sources. In this context, several plants in the Lamiaceae family, including the genus *Tetradenia* [2], have been studied for their chemical profile and potential applications in the pharmaceutical and nutraceutical sectors [3].

Among the species in this genus, *Tetradenia riparia* (Hochst.) Codd stands out. It is widely distributed in East and Tropical Africa, where it is traditionally used for various medicinal purposes [4]. In Brazil, this species was introduced as an exotic ornamental plant and is cultivated in parks, gardens, homes, and vegetable gardens [5]. Adapted to warm climates, it is popularly known by various names, such as “false myrrh,” “fog plume,” and “incense” [6]. Although published data exist, there is still insufficient evidence to classify it as a plant with potential for industrial use, and detailed investigations into its safety are lacking.

In this context, several reasons may justify the limited adoption of this medicinal plant. First, the level of knowledge about the species' bioactivity remains limited and is often based on traditional knowledge. Another reason is the lack of a robust scientific basis defining its efficacy, dosage, contraindications, and side effects. Furthermore, knowledge about its chemical composition and potential industrial applications remains scarce, hindering a comprehensive assessment of its potential benefits and safety [7].

In addition to its medicinal and ornamental uses, the species is also valued in the cosmetics and fragrance industries for its characteristic aroma [8]. Although all parts of the plant contain bioactive compounds, the leaves stand out due to their higher concentrations of volatile constituents (essential oils) and other phytochemicals [9].

Among its biological properties, the antioxidant activity of *T. riparia* has been highlighted in recent studies [10], encouraging further investigation into its potential applications in different sectors. For this species to be considered a viable source of bioactive compounds, safety studies are essential, particularly toxicological evaluations that support its safe use. Research integrating chemical composition with biological effects is therefore crucial to better understand its potential and guide future applications.

In this context, the present study provides a comprehensive and integrated evaluation of *T. riparia* leaves by combining targeted phenolic profiling using HPLC‐ESI‐QqQ‐MS/MS, antioxidant activity assessment, and in vitro toxicological analyses. This integrated approach addresses gaps in the current literature, particularly regarding the quantitative characterization of phenolic compounds and their association with biological activity. Accordingly, this study aimed to characterize the phenolic content, antioxidant activity, phytochemical diversity, and toxicological potential of *T. riparia leaves*, using aqueous extracts prepared from lyophilized leaves for chemical, antioxidant, and toxicological analyses and lyophilized plant material for evaluations of proximate composition and fatty acid profile.

## 2 Materials and Methods

### 2.1 Plant Material and Collection

Samples of *Tetradenia riparia* (Hochst.) Codd were collected in July 2022 at the Federal University of Santa Maria (UFSM), Rio Grande do Sul, Brazil (29°42′07.77″ S, 53°43′16.07″ W). The plant material was collected in the vegetative stage, which precluded the preparation and deposition of a herbarium voucher specimen. The taxonomic identification was performed based on comparison with a voucher specimen of the plant deposited in the herbarium of the Botanical Garden of UFSM (accession number JBSM1460) under the supervision of the technical staff of the Botanical Garden. Access to genetic heritage was registered in the Brazilian National System for the Management of Genetic Heritage and Associated Traditional Knowledge (SisGen) under the registration number ABE24F8.

### 2.2 Preparation of Aqueous Extract

Aqueous extracts were prepared by ultrasound‐assisted extraction (UAE), following Liu et al. [11] with slight modifications. One gram of lyophilized and milled *T. riparia* leaves was mixed with 25 mL of distilled water in 50 mL Falcon tubes and subjected to sonication (UltraCleaner 1600A, Unique, Brazil) at room temperature (approximately 25 °C) for 20 min. The extracts were centrifuged (i2206, Fanem Excelsa, Brazil) at 3000 rpm for 5 min, and the supernatant was transferred to volumetric flasks and adjusted to 25 mL with distilled water. This extraction step was repeated three additional times, and all supernatants were pooled after evaluation of phenolic content. The combined extract was filtered through a PTFE membrane (0.22 µm pore size) and stored at –20 °C until analysis.

For toxicological evaluations (cytotoxicity and genotoxicity assays), the lyophilized plant material was used directly rather than an aqueous extract in order to assess the biological effects of the whole plant matrix without selective extraction.

### 2.3 Polyphenol Content Analysis and Antioxidant Capacity

Total phenolic content was determined using the Folin–Ciocalteu method [12], adapted to a microplate format. Absorbance was measured at 760 nm after 2 h of reaction in 96‐well plates. Gallic acid (0.0109–0.0763 mg/L) was used to construct the calibration curve, and results were expressed as mg gallic acid equivalents (GAE) per 100 g of dry plant material.

Antioxidant capacity was assessed by the oxygen radical absorbance capacity (ORAC) assay following Ou et al. [13], Huang et al. [14], and Dávalos et al. [15]. Extracts (25 µL) or Trolox standards were incubated with fluorescein (0.081 µmol/L) at 37 °C for 10 min, followed by the addition of 2,2′‐azobis(2‐amidinopropane) dihydrochloride (AAPH) (0.152 µmol/L). Fluorescence decay was monitored for 90 min (excitation 485 nm, emission 528 nm), and results were expressed as mmol Trolox equivalents (TE) per 100 g of dry plant material.

### 2.4 Separation, Identification, and Quantification of Phenolic Compounds by LC–ESI–QqQ–MS/MS

Phenolic compounds were analyzed using high‐performance liquid chromatography coupled to a triple quadrupole mass spectrometer (LCMS 8045, Shimadzu, Japan) equipped with an electrospray ionization (ESI) source in negative mode and a diode array detector [16]. The aqueous extract was purified by solid‐phase extraction (SPE) on C18 cartridges (Phenomenex, USA) and eluted with methanol. Separation was achieved on a Zorbax RRHD Eclipse XDB‐C18 column (2.1 × 150 mm, 1.8 µm) at 35 °C, with a flow rate of 0.2 mL/min and an injection volume of 10 µL. The mobile phase consisted of water with 0.1% formic acid (A) and methanol (B) under a linear gradient from 0% to 100% B over 60 min.

Detection was performed under optimized MS conditions: interface voltage −3.5 kV, nebulizing gas 2 L/min, drying gas 4 L/min, and interface temperature 350 °C. Identification and quantification were based on calibration with authentic standards, and method validation followed Farias et al. [17]. The limits of detection (LOD) and quantification (LOQ) for all polyphenols analyzed are presented in Table 1.

**TABLE 1:** Limits of detection (LOD) and quantification (LOQ) for polyphenols analyzed by HPLC‐DAD‐QqQ‐MS/MS.

Compound (mg/L)	LOD	LOQ
Protocatechuic acid	0.00238	0.00500
4‐Hydroxybenzoic acid	0.00045	0.00150
Chlorogenic acid	0.00099	0.00331
Caffeic acid	0.00064	0.00215
Ellagic acid	0.01819	0.03300
Vanillic acid	0.00089	0.00297
Syringic acid	0.00115	0.00382
p‐Coumaric acid	0.00176	0.00500
Ferulic acid	0.00249	0.00500
Sinapic acid	0.00122	0.00406
Schaftoside	0.00431	0.01100
Tricin	0.00407	0.00500
Orientin	0.02393	0.03300
Rutin	0.00202	0.00500
Quercetin 3‐glucoside	0.00159	0.00500
Kaempferol	0.00126	0.00421
Kaempferol 3‐rutinoside	0.00126	0.00421
Kaempferol 3‐glucoside	0.00126	0.00421
Hesperidin	0.00265	0.00500
Quercitrin	0.00161	0.00500

### 2.5 Genotoxicity Modulation Assay

Genotoxic effects of *T. riparia* were assessed using a non‐cellular DNA interaction assay adapted from Cadoná et al. [18]. Lyophilized leaf material, directly suspended in phosphate‐buffered saline, was tested at concentrations of 1, 5, 10, 50, 100, and 500 µg/mL against double‐stranded calf thymus DNA (dsDNA). DNA integrity was evaluated by fluorescence using PicoGreen dye, which binds selectively to intact dsDNA. A decrease in fluorescence relative to the control indicated DNA fragmentation. Measurements were performed in 96‐well microplates at excitation and emission wavelengths of 480 and 520 nm, respectively, using a microplate spectrofluorometer (Hidex Sense, Finland).

### 2.6 Preparation of HFF‐1 Cell Lines

Cytotoxicity and cell proliferation were assessed using commercial human fibroblast cells (HFF‐1, SCRC‐1041 or RRID: CVCL_3285), produced and obtained by the American Type Culture Collection (ATCC‐USA). For toxicological evaluations, aqueous extracts prepared from lyophilized leaves of *T. riparia* were used in all assays. Cells were maintained in Dulbecco's Modified Eagle Medium (DMEM) supplemented with 20% fetal bovine serum, 1% penicillin–streptomycin, and 1% amphotericin B at 37 °C in a humidified atmosphere with 5% CO_2_. For assays, cells were seeded in 96‐well plates (1 × 10^4^ cells/mL) and treated with the aqueous extract of *T. riparia* leaves diluted in culture medium (DMEM) at concentrations of 5, 10, 50, 100, and 500 µg/mL. Cell viability was measured after 24 h and proliferated after 72 h using the MTT and Neutral Red (NR) uptake assays.

#### 2.6.1 MTT Assay (3‐(4,5‐Dimethylthiazol‐2‐yl)‐2,5‐Diphenyltetrazolium Bromide)

The MTT assay followed Barbisan et al. [19] with slight modifications. The reagent was prepared in phosphate‐buffered saline (PBS) at 5 mg/mL and added to each well, followed by incubation for 3 h at 37 °C. The resulting formazan crystals were solubilized in dimethyl sulfoxide (DMSO), and absorbance was measured at 570 nm using a SpectraMax i3 Multi‐Mode microplate reader (Molecular Devices, USA). Absorbance values, directly proportional to the number of metabolically active cells, were used to estimate cell viability and proliferation.

#### 2.6.2 NR Analysis

The NR uptake assay was conducted according to Repetto et al. [20]. After treatment, cells were incubated with NR solution (50 µg/mL) for 3 h, after which the dye was removed, and the incorporated NR was extracted using a desorption solution. Absorbance was read at 540 nm on the same microplate reader. This method assesses lysosomal activity as an indicator of cell membrane integrity. Therefore, higher absorbance values correspond to greater cell viability and lower cytotoxic potential of the tested samples.

### 2.7 Proximate Chemical and Fatty Acid Profile Analysis

The leaves of *T. riparia* were carefully cleaned to remove debris and extraneous material, coarsely ground, and freeze‐dried (LS3000, Terroni, Brazil). The dried material was then processed in a food‐grade multiprocessor to obtain a fine homogeneous powder, which was used for all subsequent analyses. Proximate composition analyses were performed on the freeze‐dried samples in triplicate, following AOAC [21] guidelines. Moisture content (MC) was determined by drying in an oven at 105 °C until constant weight (TE‐393/180L, Tecnal, Brazil). Protein content (PC) was determined using the Kjeldahl method for nitrogen content (TE‐036/1, Tecnal, Brazil), with a conversion factor of 5.75. Ether extract (EE) was measured with a Soxhlet apparatus (MA‐487, Marconi, Brazil) using petroleum ether for 4 h, while ash content (AC) was assessed by incineration in a muffle furnace (Q318M, Quimis, Brazil) at 550 °C for 5 h. The fiber fraction was determined as neutral detergent fiber (NDF) according to Van Soest et al. [22]. Non‐fibrous carbohydrates (or non‐NDF) were calculated as the difference between 100 and the sum of MC, AC, PC, EE, and NDF contents. The total caloric value was obtained by summing the calories produced by the metabolism of major nutrients, with results expressed as kcal/100 g.

For fatty acid profile analysis, the lipid fraction was extracted following Bligh and Dyer [23], and esterification and quantification were conducted according to Hartman and Lago [24]. Fatty acid methyl esters (FAMEs) were analyzed using a gas chromatograph (Agilent 6890, Agilent Technologies, USA) equipped with a flame ionization detector (FID) and a DB‐23 capillary column (60 m × 0.25 mm i.d., 0.25 µm film thickness). The injector and detector temperatures were maintained at 250 °C, operating in split mode (50:1) with nitrogen as the carrier gas (15 psi). The oven temperature was programmed as follows: 50 °C for 1 min, raised to 185 °C at 15 °C/min, to 195 °C at 0.5 °C/min, and finally to 230 °C at 15 °C/min, held for 5 min. Fatty acids were identified by comparison with certified FAME standards, and results were expressed as the relative percentage of total fatty acids. Only peaks with a signal‐to‐noise ratio ≥ 10:1 were considered to ensure accurate quantification and analytical reliability.

### 2.8 Statistical Analysis

Data normality was assessed using the Shapiro–Wilk test (*p* > 0.05), and variance homogeneity was examined via the Bartlett's test (*p* > 0.05). All experiments were conducted in triplicate, with results analyzed using R (RStudio) and GraphPad Prism version 8.0. One‐way ANOVA followed by Dunnett's or Tukey's *post hoc* test, as appropriate, was performed, considering *p* < 0.05 as statistically significant.

### 2.9 Results and Discussion

#### 2.9.1 Total Phenolic Content and Antioxidant Capacity of *T. riparia* Extract

**FIGURE 1:**
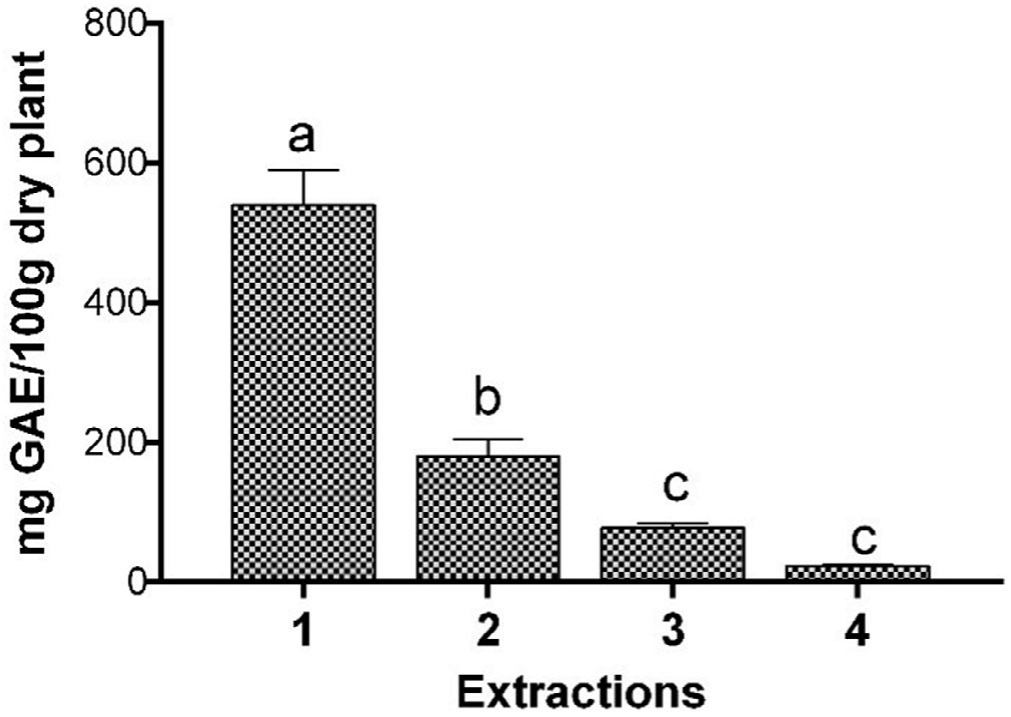
Total phenolic content (TPC) of aqueous extracts of *T. riparia* obtained by exhaustive extraction in four sequential stages. Data are expressed as mean ± standard deviation (SD) of three independent experiments (*n* = 3). Statistical analysis was performed using one‐way ANOVA followed by Tukey's post hoc test. Different lowercase letters indicate statistically significant differences (*p* < 0.05).

The total phenolic content of *T. riparia* obtained from exhaustive extraction in four sequential stages is shown in Figure 1. The results indicated that the first extraction concentrated the majority of phenolic compounds, while subsequent extractions showed a progressive decrease in their content.

The study evaluated the antioxidant activity of the crude leaf extract and fractions of *T. riparia* [25]. After dilution with methanol for analysis, the authors observed that one of the fractions, prepared with ethyl acetate–methanol (8:2), exhibited the highest total phenolic content, with 181.97 µg gallic acid/mg of sample, corresponding to 18.197 mg GAE/100 g. This value, however, is considerably lower than that found in the present study.

After combining the four extract solutions, the total phenolic content and antioxidant capacity of the resulting unified extract were evaluated (Table 2). The aqueous extract exhibited high total phenolic content and strong antioxidant activity, as determined by the ORAC assay. To our knowledge, the evaluation of phenolic compounds in sequential extractions, along with the assessment of antioxidant activity in the combined extract, has not been previously reported for this species. These results corroborate previously reported data for the species. Amoo et al. [26] reported total phenolic contents ranging from 6.1 to 7.2 mg GAE/g dry weight in fresh and long‐term stored leaves, while Fernandez et al. [25] observed that an ethanolic leaf extract and its chromatographic fractions showed a clear relationship between phenolic content and antioxidant activity (DPPH and FRAP assays), with enriched fractions reaching the highest levels. These findings are consistent with the present study and reinforce the role of phenolic compounds in the antioxidant activity of the plant, despite differences in solvents, extraction methods, and antioxidant assays.

**TABLE 2:** Total phenolic content and antioxidant capacity of combined extracts of *T. riparia* leaves.

	TPC (mg GAE/100 g)	ORAC (mmol TE/100 g)
Combined extracts	707.30 ± 15.61	24.07 ± 2.58

Variations among studies may be attributed to differences in extraction procedures, time and temperature conditions, or the solubility of phenolic compounds in different solvents [27]. Overall, the results indicate that *T. riparia* leaves are a rich source of phenolic compounds and exhibit substantial antioxidant potential, supporting their relevance for bioactive and industrial applications.

#### 2.9.2 Phenolic Profile by HPLC‐ESI‐QqQ‐MS/MS

The following phenolic compounds were identified and quantified: protocatechuic acid, 4‐hydroxybenzoic acid, caffeic acid, ellagic acid, vanillic acid, syringic acid, and *p*‐coumaric acid, all belonging to the phenolic acid class. The flavonoids tricin, orientin, kaempferol 3‐rutinoside, kaempferol 3‐glucoside, and luteolin were also identified. In addition, ferulic acid, rutin, and quercetin 3‐glucoside were detected. However, their concentrations were below the LOQ of the method and therefore could not be quantified. The LOD and LOQ for all analyzed compounds are presented in Table 1.

The identification of 15 phenolic compounds in the present study (Table 3), including both phenolic acids and flavonoids, reinforces and expands upon previous reports in the literature. Studies that employed UHPLC‐ESI/qTOF‐based approaches also detected several of the same compounds in different parts of *T. riparia*, such as luteolin, protocatechuic acid, p‐coumaric acid, caffeic acid, ferulic acid, and 4‐hydroxybenzoic acid [28, 29]. To the best of our knowledge, this study is the first to report the targeted quantitative determination of individual flavonoids and phenolic acids in an aqueous extract of *T. riparia* leaves.

**TABLE 3:** Quantification of phenolic compounds by LC‐QqQ‐MS/MS using MRM of standards in *T. riparia* leaves (mg/100 g of dry plant material).

Class	Compounds	RT (min)	Transitions^*^ (*m/z*)	Pre‐bias (Q1) (V’)	Collision energy (V’)	Pre‐bias (Q3) (V’)	Mean ± SD
Phenolic acids	Protocatechuic acid	10.615	153.10–09.05	13	15	12	0.095 ± 0.007
			153.10–08.00	13	23	19	
			153.10–90.80	13	26	10	
	4‐Hydroxybenzoic acid	14.597	137.20–93.00	29	16	16	0.185 ± 0.003
			137.20–65.05	29	30	24	
			137.20–41.10	30	49	18	
	Caffeic acid	19.499	179.10–135.15	13	16	14	1.271 ± 0.384
			179.10–134.05	14	26	24	
	Ellagic acid	24.734	300.90–284.00	20	30	29	0.360 ± 0.017
			300.90–145.15	20	37	25	
			300.90–229.10	20	28	24	
	Vanillic acid	18.800	167.15–152.00	15	15	27	0.718 ± 0.087
			167.15–108.05	13	19	12	
			167.15–123.15	15	14	14	
	Syringic acid	21.070	197.00–182.15	18	14	19	0.016 ± 0.001
			197.00–123.05	14	24	24	
			197.00–167.10	14	19	30	
	*p*‐Coumaric acid	24.469	163.10–119.10	15	15	13	0.114 ± 0.051
			163.10–93.05	15	32	18	
			163.10–117.15	13	33	22	
	Ferulic acid	25.698	193.00–134.05	14	17	14	nq
			193.00–178.15	14	14	19	
			193.00–149.15	14	13	16	
Flavonoids	Luteolin	27.219	562.90–353.05	28	37	17	0.284 ± 0.032
			562.90–383.05	28	36	26	
			562.90–296.95	30	53	30	
	Tricin	30.217	490.90–329.05	25	27	23	0.798 ± 0.116
			490.90–314.00	25	39	22	
			490.90–299.00	25	45	29	
	Orientin	25.980	446.90–326.95	23	23	23	1.226 ± 0.157
			446.90–357.05	24	21	26	
			446.90–299.05	24	37	20	
	Rutin	25.439	609.10–300.00	22	37	30	nq
			609.10–301.00	22	32	15	
			609.10–271.00	22	55	28	
	Quercetin 3‐glucoside	25.919	463.20–300.00	17	27	30	nq
			463.20–301.10	13	23	20	
			463.20–271.10	17	44	28	
	Kaempferol 3‐rutinoside	26.020	593.00–285.00	22	32	30	0.098 ± 0.010
			593.00–284.00	26	39	29	
			593.00–255.05	22	55	27	
	Kaempferol 3‐glucoside	26.004	447.00–283.90	11	28	30	0.121 ± 0.038
			447.00–254.95	11	40	27	
			447.00–227.05	12	49	24	

*Note*: RT: Retention time; m/z: mass/charge; V’: voltage. The standards without a calibration curve were quantified with the nearest structural standards. nq: not quantifiable. Mean value ± standard deviation (*n* = 3).

* One transition used for quantification, other transitions for identification.

In this context, the present work contributes additional data by identifying and quantifying a broader range of phenolic compounds, including tricin, orientin, and kaempferol derivatives, in aqueous extracts. These findings not only corroborate earlier reports but also highlight the chemical complexity and potential of *T. riparia* as a source of bioactive phenolics. The chemical characterization obtained by HPLC‐ESI‐QqQ‐MS/MS indicates that the aqueous leaf extract contains compounds widely associated with antioxidant mechanisms, including hydroxybenzoic and hydroxycinnamic acids, as well as flavone and flavonol derivatives. Compounds such as caffeic acid, p‐coumaric acid, protocatechuic acid, ellagic acid, luteolin, and kaempferol derivatives are known for their ability to donate hydrogen atoms and stabilize reactive species, which is directly relevant to the ORAC assay. In this context, the phenolic profile identified in the present study is consistent with the total phenolic content and antioxidant capacity observed. Similar relationships between phenolic composition and radical scavenging activity have been reported by Moazzen et al. [30], supporting the association between the identified phenolic compounds and the antioxidant activity observed in the present study.

#### 2.9.3 Potential Genotoxic Effects

**FIGURE 2:**
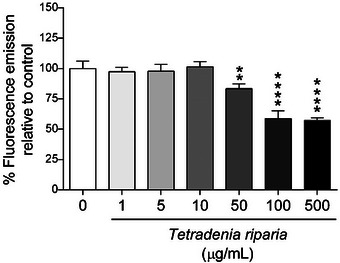
Evaluation of the genotoxic effects of different concentrations of *T. riparia* leaf aqueous extract using the genomodifier capacity (GEMO) assay. Data are expressed as mean ± SD of three independent experiments (*n* = 3). Statistical analysis was performed using one‐way ANOVA followed by Dunnett's post hoc test, considering *p* < 0.05 as statistically significant. Symbols indicate levels of significance: ** *p* ≤ 0.01 and **** *p* ≤ 0.0001 compared to the negative control.

According to the results of the genomodifier capacity assay (GEMO) (Figure 2), the concentrations of 1, 5, and 10 µg/mL showed no statistically significant difference compared to the negative control group, indicating that at these concentrations, the aqueous extract did not cause significant DNA degradation and showed no genotoxic activity.

In contrast, concentrations above 50 µg/mL showed a statistically significant difference, suggesting that at higher doses, the extract may cause greater DNA fragmentation, which is indicative of genotoxic effects.

#### 2.9.4 Cell Viability Assays

**FIGURE 3:**
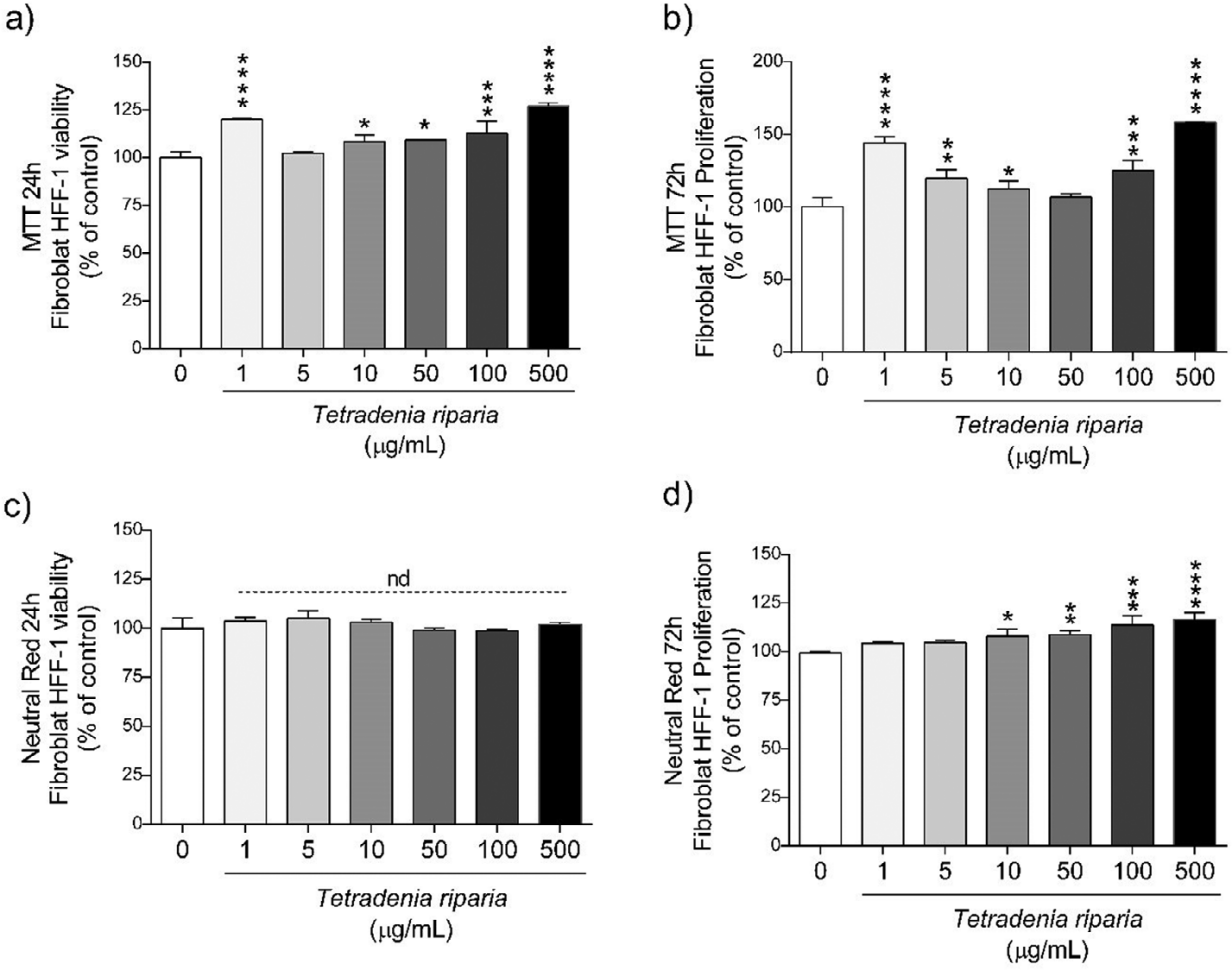
Cell viability (24 h) and proliferation capacity (72 h) of HFF‐1 cells after exposure to different concentrations of *T. riparia* leaf aqueous extract. Data are expressed as mean ± SD of three independent experiments (*n* = 3). Statistical analysis was performed using one‐way ANOVA followed by Dunnett's post hoc test, with *p* < 0.05 considered statistically significant. Differences relative to the control are indicated as follows: *p ≤ 0.05; **p ≤ 0.01; ***p ≤ 0.001; ****p ≤ 0.0001.

In the cell viability test, performed using normal HFF‐1 cell line, it was observed that after 24 h (Figure 3a), all concentrations showed significantly higher values compared to the control, indicating a positive effect on cell viability. After 72 h (Figure 3b), the pattern remained similar to that observed at 24 h, with all concentrations maintaining high cell viability, suggesting a sustained positive effect. These results indicate that the concentrations tested were not cytotoxic, meaning they did not cause cell death. The effect observed at both 24 and 72 h may reflect increased cell proliferation or cellular adaptation to the treatment.

In the NR assay, no statistical difference was observed compared to the control after 24 h of exposure (Figure 3c), suggesting that, at this stage, there was no significant effect on cell viability. However, after 72 h (Figure 3d), the concentrations of 1 and 5 µg/mL showed no significant difference compared to the control, while the concentrations of 10, 50, 100, and 500 µg/mL showed statistically significant differences, with values higher than the control. This suggests that higher concentrations may have beneficial effects on cell viability, increasing cell survival compared to the control, possibly due to a positive or adaptive cellular response.

The MTT and NR assays demonstrated that the *T. riparia* leaf aqueous extract was not cytotoxic at the concentrations tested. On the contrary, concentrations of 10, 50, 100, and 500 µg/mL significantly increased cell viability after 72 h, suggesting a stimulatory effect on cellular metabolism.

Given the high phenolic content and antioxidant capacity of *T. riparia*, potential interference in redox‐based assays, such as MTT, was acknowledged, as phenolic compounds can directly reduce tetrazolium salts, thereby artificially increasing absorbance values [31]. To minimize this concern, control assays were performed by incubating the extract with MTT in the absence of cells, with no detectable formazan formation. In addition, cell viability was independently confirmed using the NR uptake assay, a non–redox‐based method. The concordance between MTT and NR supports the reliability of the viability data and indicates that the observed effects are unlikely to be due to assay interference.

Genotoxicity analyses revealed that no significant DNA damage was observed at concentrations of 1, 5, and 10 µg/mL. However, DNA fragmentation occurred at concentrations ≥ n50 µg/mL, indicating genotoxic effects at higher doses (Figure 2). These findings suggest a dual effect: while moderate doses may enhance cell viability and proliferation, elevated concentrations can compromise genomic integrity. To the best of our knowledge, this is the first report evaluating these effects in HFF‐1 human fibroblasts.

Previous studies on the essential oil of *T. riparia* reported high cytotoxic activity against tumor cell lines such as SF‐295, HCT‐8, and MDA‐MB‐435 using the MTT assay [32], whereas another investigation observed reduced proliferation in B16F10 and HT29 cells, although without sufficient potency to support its development as an anticancer agent [33]. While those studies focused on tumor lines and essential oil, the present study provides novel insights into the effects of the crude extract on non‐tumor human fibroblasts.

These findings are in agreement with previous studies evaluating the genotoxic potential of this species using different experimental models. Okem et al. [34], using the Ames test with *Salmonella typhimurium*, reported no direct mutagenic activity for leaf extracts at the tested concentrations, although the authors emphasized that the absence of mutagenicity in bacterial systems does not guarantee absolute safety. Similarly, Taylor et al. [35] observed no significant genotoxic effects of methanol–water and dichloromethane leaf extracts in human lymphocytes assessed by micronucleus and comet assays, reinforcing the indication of low genotoxic risk at moderate exposure levels.

More recently, Ndayambaje et al. [10] demonstrated the absence of acute and sub‐acute oral toxicity in vivo for a hydroalcoholic leaf extract, even at high doses, supporting the relative safety of this species under controlled conditions. Taken together, these studies and the present results suggest that the *T. riparia* leaf aqueous extract exhibits low genotoxic potential at lower concentrations, while higher doses may induce DNA damage, highlighting the importance of dose‐dependent evaluation and careful consideration of exposure levels.

#### 2.9.5 Proximate Chemical, Energy Value, and Fatty Acid Profile Analysis

The proximate composition refers to the main ingredients of the food, such as protein, fat, ash, moisture, fiber, and carbohydrates. The results of the chemical composition of the dried leaves of *T. riparia* are presented in Table 4.

**TABLE 4:** Nutritional components, energy value, and fatty acid profile of *T. riparia* leaves, mean value ± standard deviation (*n* = 3).

^a^Proximate composition	
Ash	12.46 ± 1.17
Crude protein*	14.14 ± 0.56
Ether extract	6.27 ± 0.18
NDFa	52.00 ± 3.12
non‐NDFa CHO	15.13
Energy (kcal)	173.51

^b^Fatty acid profile	
C12:0	0.06 ± 0.04
C13:0	0.05 ± 0.01
C14:0	0.09 ± 0.02
C16:0	18.84 ± 0.31
C16:1 n‐7	0.19 ± 0.01
C17:0	0.17 ± 0.006
C18:0	1.74 ± 0.04
C18:1 n‐9c	7.54 ± 0.11
C18:2 n‐6c	49.59 ± 0.54
C18:3 n‐3	19.63 ± 0.17
C20:0	0.71 ± 0.04
C22:0	0.96 ± 0.04
C24:0	0.42 ± 0.009
SFA	23.04 ± 0.32
MUFA	7.73 ± 0.11
PUFA	69.22 ± 0.57
PUFA/SFA	3.00
n‐6	49.59 ± 0.54
∑ n‐3	19.63 ± 0.17
n‐6/n‐3	2.52

*Note*: Nitrogen‐to‐protein conversion factor – 5.75. NDFa = neutral detergent fiber corrected for ash; non‐NDFc CHO = non‐NDFc carbohydrates. SFA (saturated fatty acids = C12:0; C13:0; C14:0; C16:0; C17:0; C18:0; C20:0; C22:0; C24:0); MUFA (monounsaturated fatty acids = C16:1 n‐7; C18:1 n‐9); PUFA (polyunsaturated fatty acids = C18:2 n‐6c; C18:3 n‐3).

^a^ Results expressed as g/100 g of dry matter.

^b^ Values expressed as percentage of the total chromatogram area.

The proximate composition revealed an AC of 12.46 g/100 g, indicating a notable mineral contribution. Crude PC was 14.14 g/100 g, suggesting relevant nutritional potential. The EE was 6.27 g/100 g, reflecting a moderate lipid presence. The NDF fraction reached 52 g/100 g, evidencing a high fiber content, while non‐fibrous carbohydrates (non‐NDF) accounted for 15.13 g/100 g. The estimated energy value was 173.51 kcal/100 g, highlighting the plant's potential as an energy source. To date, no previous studies have reported the proximate composition of *T. riparia*, emphasizing a gap in the literature regarding its nutritional characteristics.

The fatty acid profile (Table 4) showed a predominance of unsaturated fatty acids, particularly linoleic (C18:2 n‐6) and α‐linolenic (C18:3 n‐3) acids, which are essential components of the human diet [36]. These compounds play crucial roles in maintaining cell membrane integrity, modulating inflammatory responses, and supporting cardiovascular health. Among the saturated fatty acids (SFA) identified were lauric (C12:0), tridecanoic (C13:0), myristic (C14:0), palmitic (C16:0), stearic (C18:0), arachidic (C20:0), behenic (C22:0), and lignoceric (C24:0) acids. The monounsaturated fatty acid (MUFA) fraction included palmitoleic (C16:1 n‐7) and oleic (C18:1 n‐9) acids, while the main polyunsaturated fatty acids (PUFA) were linoleic (C18:2 n‐6) and α‐linolenic (C18:3 n‐3). The presence of oleic acid is noteworthy due to its recognized cardioprotective effects.

Although SFAs such as palmitic and stearic acids were detected, their moderate levels suggest a limited potential for adverse effects on cardiovascular health. The n‐6/n‐3 ratio was 2.52:1, a nutritionally favorable value, as international guidelines recommend ratios below 10:1 to maintain an adequate balance between pro‐ and anti‐inflammatory processes [37]. This proportion may contribute to the prevention of chronic conditions such as cardiovascular diseases, obesity, and metabolic disorders.

No prior studies have characterized the fatty acid composition of *T. riparia* using the analytical standard applied here. Most available research focuses on the chemical profile and biological properties of its essential oils.

Compared with other leafy plant species analyzed previously [27], these levels are comparatively high, highlighting the nutritional value of *T. riparia* leaves in terms of both proximate composition and fatty acid profile. Further investigations are warranted to evaluate the bioavailability and potential incorporation of *T. riparia* leaf‐derived products into functional foods or nutraceutical formulations.

## 3 Conclusions

This study provides the first comprehensive characterization of *T. riparia* leaves, covering total phenolic content, antioxidant capacity, phenolic composition, genotoxicity, cytotoxicity, and nutritional profile. The leaf aqueous extract exhibited a rich diversity of phenolic compounds associated with high antioxidant potential, as determined by the Folin‐Ciocalteu and ORAC assays. Genotoxicity assessment performed using the *T. riparia* leaf aqueous extract revealed no DNA fragmentation at concentrations below 50 µg/mL, indicating the absence of genotoxic effects. Similarly, cytotoxicity tests showed no toxic response and suggested increased cell viability, potentially reflecting a proliferative or adaptive effect. Proximate analysis indicated relevant nutritional value, with high protein and fiber content, while the fatty acid profile was dominated by unsaturated compounds, particularly linoleic and α‐linolenic acids, producing a favorable n‐6/n‐3 ratio. Overall, *T. riparia* combines nutritional and bioactive properties, supporting its potential applications in the pharmaceutical and nutraceutical sectors. Future studies should investigate the mechanisms underlying its bioactivities, the bioavailability of its compounds, and their efficacy using in vivo models.

## Author Contributions

**Caroline dos Santos Giuliani**: investigation, validation, writing – original draft. **Carla Andressa Almeida Farias**: investigation, methodology, data curation, formal analysis, validation. **Patrícia Kolling Marquezan**: investigation, methodology, formal analysis, validation. **Karoline Longo de Aguiar**: investigation, methodology, data curation, formal analysis. **Andreara Rodrigues dos Reis**: investigation, methodology, data curation, formal analysis. **Elize Musachio**: investigation, methodology, data curation, formal analysis. **Nathália Cardoso de Afonso Bonotto**: investigation, methodology, data curation, formal analysis. **Fernanda Barbisan**: investigation, methodology, data curation, formal analysis, validation. **Milene Teixeira Barcia**: investigation, methodology, data curation, formal analysis, validation. **Ana Lúcia Souza Silva Mateus**: formal analysis, methodology, software. **Aline Sobreira Bezerra**: conceptualization, resources, supervision, writing – revise and editing, investigation. **José Laerte Nörnberg**: conceptualization, investigation, resources, supervision, validation, writing – revise and editing.

## Conflicts of Interest

The authors declare no conflicts of interest.

## Data Availability

The data that support the findings of this study are available from the corresponding author upon reasonable request.
